# Predicting the risk of death following coronary artery bypass graft made simple: a retrospective study using the American College of Surgeons National Surgical Quality Improvement Program database

**DOI:** 10.1186/s13019-015-0269-y

**Published:** 2015-04-29

**Authors:** Paul J Chung, Timothy I Carter, Joshua H Burack, Sophia Tam, Antonio Alfonso, Gainosuke Sugiyama

**Affiliations:** 1 Department of Surgery, State University of New York Downstate Medical Center, 450 Clarkson Ave, Brooklyn, NY 11203 USA; 2 Department of Cardiothoracic Surgery, State University of New York Downstate Medical Center, Brooklyn, 11203 USA; 3 College of Medicine, State University of New York Downstate Medical Center, Brooklyn, 11203 USA

**Keywords:** Coronary artery bypass graft, Coronary artery disease, Risk model, Postoperative mortality, ACS NSQIP, Database

## Abstract

**Introduction:**

Risk models to predict 30-day mortality following isolated coronary artery bypass graft is an active area of research. Simple risk predictors are particularly important for cardiothoracic surgeons who are coming under increased scrutiny since these physicians typically care for higher risk patients and thus expect worse outcomes. The objective of this study was to develop a 30-day postoperative mortality risk model for patients undergoing CABG using the American College of Surgeons National Surgical Quality Improvement Program database.

**Material and methods:**

Data was extracted and analyzed from the American College of Surgeons National Surgical Quality Improvement Program Participant Use Files (2005–2010). Patients that had ischemic heart disease (ICD9 410–414) undergoing one to four vessel CABG (CPT 33533–33536) were selected. To select for acquired heart disease, only patients age 40 and older were included. Multivariate logistic regression analysis was used to create a risk model. The C-statistic and the Hosmer-Lemeshow goodness-of-fit test were used to evaluate the model. Bootstrap-validated C-statistic was calculated.

**Results:**

A total of 2254 cases met selection criteria. Forty-nine patients (2.2%) died within 30 days. Six independent risk factors predictive of short-term mortality were identified including age, preoperative sodium, preoperative blood urea nitrogen, previous percutaneous coronary intervention, dyspnea at rest, and history of prior myocardial infarction. The C-statistic for this model was 0.773 while the bootstrap-validated C-statistic was 0.750. The Hosmer-Lemeshow test had a p-value of 0.675, suggesting the model does not overfit the data.

**Conclusions:**

The American College of Surgeons National Surgical Quality Improvement Program risk model has good discrimination for 30-day mortality following coronary artery bypass graft surgery. The model employs six independent variables, making it easy to use in the clinical setting.

## Background

Developing risk models to predict 30-day postoperative mortality following isolated coronary artery bypass graft (CABG) has been an active area of research [1-3]. No such risk model has been developed using the American College of Surgeons National Surgical Quality Improvement Program (ACS NSQIP) database. This multi-institutional database was created to provide clinical data for improving the quality of surgical outcomes [4,5]. ACS NSQIP now obtains data from more than 525 participating hospitals covering approximately 30% of the operative volume of the United States [6,7]. Demographic, preoperative comorbidity, operative data, and 30-day postoperative morbidity and mortality data is collected in a systematic and standardized manner for use in outcomes research [8]. The objective of this study was to develop a 30-day postoperative mortality risk model for isolated CABG utilizing ACS NSQIP, simplified for clinical practice.

## Methods

Exempt status was granted from our institution’s internal review board. Data was taken from the 2005–2010 ACS NSQIP Participant Use Files, including 240 demographic, preoperative, operative, and morbidity/mortality variables. Diseases and procedures are classified using International Classification of Diseases 9 Clinical Modification (ICD-9-CM) and Current Procedural Terminology (CPT) codes.

Patients who underwent CABG using one to four vessel arterial grafts (CPT codes 33533–33536) were included in the study. These codes also include coronary artery bypass procedures using arterial grafts only, or a combination of arterial-venous grafts [9]. To focus on ischemic heart disease, only patients with a diagnosis of ischemic heart disease (ICD-9-CM codes 410–414) were included. To select for acquired heart disease, only patients 40 years and older were included. A total of 66 variables that covered demographic, comorbidity, preoperative laboratory values, previous medical/surgical interventions, and perioperative data were used in the analysis.

Univariate analysis was performed with *p* < 0.2 as the inclusion criteria. Multiple imputation was performed on continuous variables. Variables with more than 5% of values missing were not included in univariate analysis [10]. The Wilcoxon rank sum test and Pearson’s X^2^ test were used for continuous and categorical variables respectively. Stepwise backwards selection was used on the outcome of univariate analysis and candidate variables were obtained (Table 1) for multivariate logistic regression analysis. Variables with *p* < 0.05 were candidates for inclusion in the final multivariate model, however those with wide 95% confidence interval were excluded. The C-statistic was used to determine the model’s discriminative ability [11]. The bootstrap method was used to find the optimism-corrected C-statistic [12]. The Hosmer-Lemeshow statistic was used to determine goodness of fit [13]. A risk score was then created using the final multivariate statistical model. Statistical analysis was performed using R version 3.0 [14].

**Table 1:** **Results of Univariate Analysis**

	Survived Past 30 Days	Mortality Within 30 Days	
Characteristic †	*n = 2205*	*n = 49*	*P*value
Age, yr, mean (SD)	65.52 (10.1)	70.84 (8.8)	0.0002
Sex			0.2907
Male	1654 (75.0%)	33 (67.3%)	
Female	551 (25.0%)	16 (32.7%)	
Height, m, mean (SD)	1.72 (0.1)	66.77 (4.23)	0.0126
Weight, kg, mean (SD)	88.9 (20.0)	85.5 (29.7)	0.1737
BMI, mean (SD)	30.1 (6.3)	29.0 (8.4)	0.0372
Emergency			0.0087
ASA Classification			0.0712
Class I	1 (0.0%)	0 (0.0%)	
Class II	10 (0.5%)	0 (0.0%)	
Class III	661 (30.0%)	5 (10.2%)	
Class IV	1519 (69.0%)	43 (87.8%)	
Class V	10 (0.5%)	1 (2.0%)	
Dyspnea			0.0017
None	1229 (55.7%)	20 (40.8%)	
With exertion	842 (38.2%)	19 (38.8%)	
At rest	134 (6.1%)	10 (20.4%)	
History of MI	707 (32.1%)	28 (57.1%)	0.0004
History of CHF	175 (7.9%)	9 (18.4%)	0.0176
History of PCI	667 (30.2%)	19 (38.8%)	0.2602
Previous Cardiac Surgery	68 (3.1%)	4 (8.2%)	0.1120
Hypertension Requiring Medication	1820 (82.5%)	45 (91.8%)	0.1305
History of Revascularization/Amputation for PVD	101 (4.6%)	6 (12.2%)	0.0311
Rest Pain/Gangrene	14 (0.6%)	4 (8.2%)	<0.0001
Acute Renal Failure	9 (0.4%)	2 (4.1%)	0.0090
Functional Status			0.0002
Independent	2047 (92.8%)	40 (81.6%)	
Partially Dependent	129 (5.9%)	5 (10.2%)	
Totally Dependent	29 (1.3%)	4 (8.2%)	
Do Not Resuscitate	10 (0.5%)	2 (4.1%)	0.0139
Coma >24 Hours	1 (0.0%)	1 (2.0%)	0.0268
Chemotherapy Within 30 Days of Surgery	2 (0.1%)	1 (2.0%)	0.0849
Transfusion >4 Units pRBCs Within 72 Hours of Surgery	6 (0.3%)	2 (4.1%)	0.0013
Systemic Sepsis			< 0.0001
None	2094 (96.6%)	45 (91.8%)	
SIRS	66 (3.0%)	3 (6.1%)	
Sepsis	7 (0.3%)	0 (0.0%)	
Septic Shock	1 (0.0%)	1 (2.0%)	
Preoperative Sodium, mean (SD)	138.1 (3.0)	136.5 (3.4)	0.0012
Preoperative BUN, mean (SD)	18.7 (9.7)	24.0 (10.7)	< 0.0001
Preoperative Creatinine, mean (SD)	1.14 (0.8)	1.30 (0.8)	0.0026
Preoperative Hematocrit, mean (SD)	39.1 (5.1)	37.3 (5.0)	0.0191
Preoperative Albumin, mean (SD)	3.83 (0.5)	3.49 (0.7)	0.0001
Preoperative AST, mean (SD)	31.4 (30.4)	40.7 (47.8)	0.1024
Preoperative PT, mean (SD)	12.5 (2.61)	14.2 (3.6)	< 0.0001
Preoperative INR, mean (SD)	1.1 (0.1)	1.2 (0.3)	0.0045
Preoperative PTT, mean (SD)	36.6 (17.3)	41.4 (21.1)	0.0148
Units of pRBCs Given Intraoperatively, mean (SD)	1.4 (1.8)	2.7 (2.9)	0.0043
Duration Patient in Room, min, mean (SD)	345.3 (83.6)	373.2 (111.0)	0.1329

SD = standard deviation, ASA = American Society of Anesthesiologists, AST = aspart aminotrasnferase, BMI = body mass index, BUN = blood urea nitrogen, CHF = congestive heart failure, INR = international normalized ratio, MI = myocardial infarct, PCI = percutaneous coronary intervention, PCS = previous cardiac surgery, pRBC = packed red blood cell, PT = prothrombin time, PTT = partial thromboplastin time, PVD = peripheral vascular disease, SIRS = systemic inflammatory response syndrome.

† Missing values excluded from calculations.

## Results

From 2005–2010, a total of 2254 cases fitting the inclusion criteria were found from a total of 4317 CABGs recorded during that time period. Most patients were male (74.8%, n = 1687), with a median age of 66.0 years. The incidence of mortality within 30-days of surgery was 2.2% (n = 49), involving mostly males (67.3%, n = 33), and a median age of 70.8 years.

The univariate analysis results are shown in Table 1. Although a cutoff of *p* < 0.2 was chosen, current literature supported the addition of sex, body mass index (BMI), and previous percutaneous coronary intervention (PCI) to multivariate analysis [2,15,16]. In multivariate analysis, previous PCI was found to be marginally significant with *p* = 0.0648, however it was kept because the C-statistic of the final model decreased from 0.773 to 0.762, suggesting its importance. Of note, history of ischemic rest pain/gangrene, renal failure, transfusions of four or more units of red blood cells, and do not resuscitate status were not included in the final model because of wide 95% confidence intervals. Duration of operation was significant but excluded, without detriment to the model’s discriminative ability, to ensure all variables could be obtained preoperatively. The final model contained only six variables (Table 2).

**Table 2:** **Results of Multivariate Analysis**

Variable*	Odds ratio	95% CI	*P*value	β coefficient
Age	2.28	1.43 - 3.62	0.0005	0.0549
Dyspnea with Moderate Exertion	1.39	0.73 - 2.66	0.3174	0.3306
Dyspnea at Rest	2.99	1.30 - 6.87	0.0101	1.0937
History of MI	2.52	1.38 - 4.62	0.0027	0.9246
Previous PCI	1.76	0.97 - 3.21	0.0648	0.5655
Preoperative Sodium	0.64	0.46 - 0.88	0.0062	−0.1135
Preoperative BUN	1.20	1.03 - 1.40	0.0194	0.0204

*Odds ratios for continuous variables were calculated comparing first and third quartile values.

Age: 60 years : 74 years.

Preoperative Sodium: 136 mmol/L: 140 mmol/L.

Preoperative BUN: 13 mg/dL: 22 mg/dL.

MI = myocardial infarct, PCI = percutaneous coronary intervention, BUN = blood urea nitrogen.

**Figure 1:**
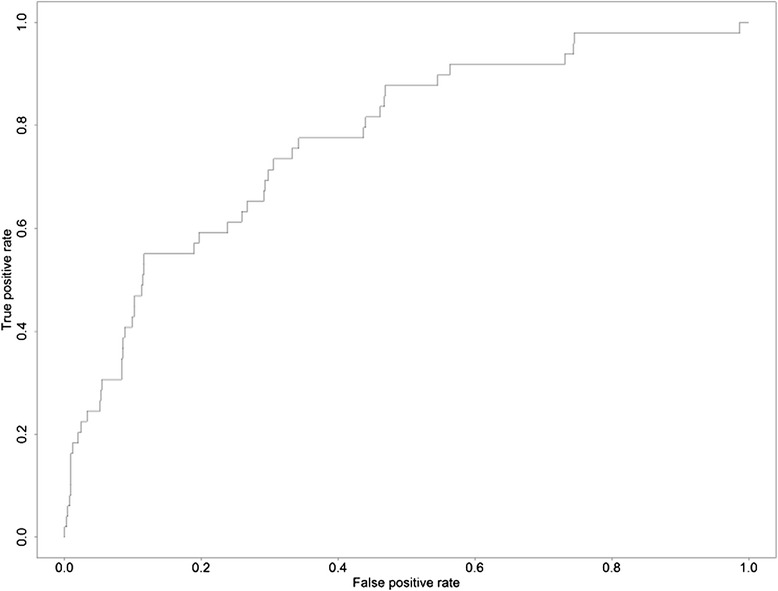
Receiver Operator Characteristic of the ACS-NSQIP 30-Day Postoperative Risk Model.

The C-statistic for the final model was 0.773, demonstrating good discriminative ability (Figure 1). The optimism-corrected C-statistic found using the bootstrap method was 0.750. The Hosmer-Lemeshow statistics also suggested that the model did not over fit the data (*p* = 0.675).

**Figure 2:**
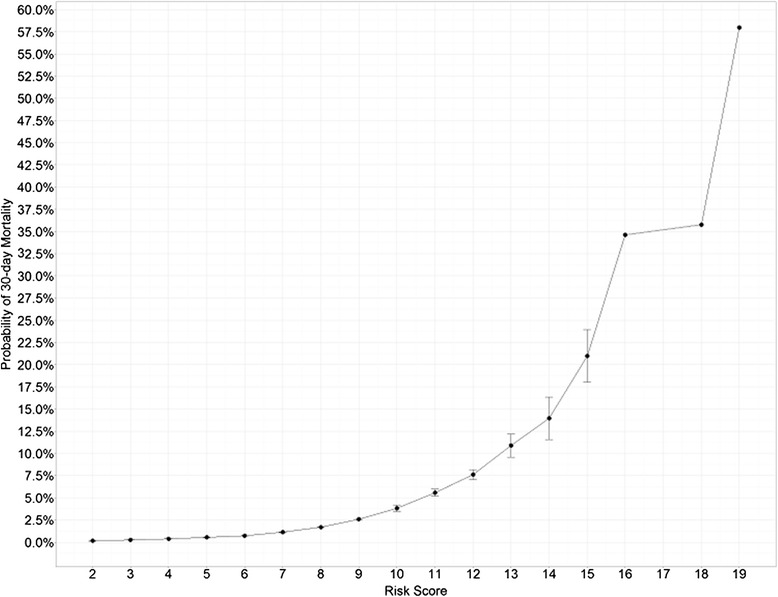
Calculated Probability of 30-Day Mortality Following CABG vs Risk Score.

A risk score was created using the β coefficients of logistic regression model. The risk score ranged from 1 to 23 (Table 3). The lowest observed risk score from the data was two while the highest was 19 (Figure 2). The probability of 30-day mortality ranged from 0.14% for patients with a score of two, to 58.0% for those with a score of 19. The mean probability of mortality was 2.2%, the median was 1.3%, and the standard deviation was 3.0%. A score of eight or less corresponded to a predicted mortality less than the mean, which included 1860 patients (82.5%), while a score of nine approximately corresponded to the mean probability of mortality. Twenty-one patients (0.9%) had a predicted mortality greater than two standard deviations from the mean probability (8.2%), which corresponded to a risk score of 12 or greater.

**Table 3:** **Risk Score for 30-Day Mortality Following Coronary Artery Bypass Graft**

Risk Factors	Score
Age	
40-49 years	1
50-59 years	2
60-69 years	3
70-79 years	4
>80 years	5
Dyspnea	
None	0
Moderate Exertion	1
At Rest	3
History of MI	
No	0
Yes	2
Previous PCI	
No	0
Yes	1
Preoperative BUN	
< 30	0
≥ 30	2
Preoperative Sodium	
> 145 mEq/L	0
140 - 145 mEq/L	1
135 - 139 mEq/L	3
130 - 134 mEq/L	4
125 - 129 mEq/L	6
120 - 124 mEq/L	7
115 - 119 mEq/L	9
< 115 mEq/L	10

Lowest score = 1.

Highest score = 23.

*MI = myocardial infarct.*

*PCI = percutaneous coronary intervention.*

*BUN = blood urea nitrogen.*

## Discussion

We present a simple risk score to estimate 30-day mortality following isolated CABG with discrimination comparable to other major risk calculators in cardiac surgery [1-3]. To assess outcomes and to provide patients and families with realistic predictions of operative risk, the need for simple preoperative risk calculators remains ever present. With outcome data publically available, risk assessment tools are necessary for cardiothoracic surgeons, who are coming under increased scrutiny [17]. Risk scores can “level the playing field,” to fairly assess outcomes for surgeons who care for higher risk patients and therefore expect worse outcomes.

The ACS NSQIP model shares many similarities with other risk models such as EuroSCORE [1], the New York Risk Score [3,18], and the Society of Thoracic Surgeons (STS) 2008 Cardiac Surgery Risk Model [2]. Shared variables include age, previous MI, PVD, renal failure, hemodynamic state and ejection fraction (EF). An ad hoc committee concluded that seven core variables should be present in any database reporting risk-adjusted outcomes for CABG which include age, sex, previous heart operation, EF, percent stenosis of left main coronary artery, number of major coronaries with greater than 70% stenosis and level of acuity [15]. In the STS model, 78% of the variance is explained by eight of the most important variables, which include age, surgical acuity, reoperative status, creatinine level, dialysis, shock, chronic lung disease, and EF [19]. While not a specialty database tailored to specific procedures, the ACS NSQIP database includes all of these core variables except for EF and other cardiac variables. Our model includes several variables listed in the consensus statement and other CABG datasets (age, previous MI, pervious PCI) while also finding ischemic rest pain/gangrene (a proxy for peripheral vascular disease), renal failure, and preoperative transfusion (a proxy for preoperative anemia) significant, but ultimately excluded due to their high confidence intervals. Preoperative anemia, while not a core variable, is a known risk factor for mortality following CABG [20].

Estimating risk involves using discrete markers as surrogates for the extent of cardiac disease. Core variables attempt to encapsulate the extent of a patient’s cardiovascular disease, the impact of that disease on end organ function, and ultimately reserve. BUN addresses not only the degree of renal dysfunction, but also the quality and frequency of hemodialysis—important since the impact of uremia on cardiac function is well known [21]. We argue that in recent times, previous PCI might be more valid than previous cardiac surgery, in qualifying the severity of cardiac disease. Finally, hyponatremia is increasingly recognized as a predictor of postoperative morbidity and mortality following both cardiac and general surgery [22]. Crestanello et al. [23] found preoperative hyponatremia as a risk factor for increased long-term morbidity, prolonged hospitalization, and mortality in patients undergoing cardiac surgery.

The ACS NSQIP model includes variables not yet included in specialty databases, but are known risk factors for mortality following CABG, such as those covering liver disease [2]. This model covers a large subset of core variables common to the major risk models, without sacrificing discriminative ability. We argue that using a non-specialty database, while controversial, may prove beneficial to a field where risks models all share similar variables and “room for improvement for discriminating adverse outcomes may be limited [24].”

There are several limitations to this study. First, this is a retrospective study with data obtained by dedicated staff in a systematic fashion. Second, sample size was another limitation as there were fewer patients available to create the risk model compared to other specialty databases. A likely reason as to why there were fewer CABG cases during the 6 year period over which the study is employed is that sites submitting data to an established cardiac database could request for exemption for submission of cardiac subspecialty data [25]. The paucity of data is perhaps secondary to the influence of other specialty databases, such as the STS ACSD [19]. Third, cardiac-related variables were not available for inclusion in the final model. As a more generalized database, ACS NSQIP does not record all variables recommended in the consensus statement on the development of risk calculators [15]. Despite these limitations, the ACS NSQIP risk model shows a discriminative ability on par with other available risk models.

## Conclusion

In conclusion, our risk model provides cardiothoracic surgeons with a simple to use tool for assessing risk of mortality following CABG using six preoperative, independent variables that can be assessed with history, physical exam and a serum chemistry panel. Finally our study demonstrates the versatility of the ACS NSQIP database.

## Abbreviations

CABG: Coronary Artery Bypass Graft
ACS NSQIP: American College of Surgeons National Surgical Quality Improvement Program
IRB: Internal Board Review
ICD: International Classification of Disease
CPT: Current Procedural Terminology
ROC: Receiver Operating Characteristic
BMI: Body Mass Index
